# Supplementary material for: Modeling glioblastoma heterogeneity as a dynamic network of cell states
Source: Mol Syst Biol. 2021 Sep 16;17(9):e10105. doi: 10.15252/msb.202010105 (PMC8444284; doi:10.15252/msb.202010105)
Supplement: Supplementary file 6 — Source Data for Figure 5 [file MSB-17-e10105-s004.zip › Figure5A_sourcedata/GSEA_3071/hallmarks_stateII.GseaPreranked.1623742536692/HALLMARK_EPITHELIAL_MESENCHYMAL_TRANSITION.html]

Details for gene set HALLMARK\_EPITHELIAL\_MESENCHYMAL\_TRANSITION[GSEA]

|  || Dataset | state23071 |
| Phenotype | NoPhenotypeAvailable |
| Upregulated in class | na\_pos |
| GeneSet | HALLMARK\_EPITHELIAL\_MESENCHYMAL\_TRANSITION |
| Enrichment Score (ES) | 0.3144737 |
| Normalized Enrichment Score (NES) | 1.8528714 |
| Nominal p-value | 0.0 |
| FDR q-value | 0.0104068555 |
| FWER p-Value | 0.064 |
Table: GSEA Results Summary

  

Fig 1: Enrichment plot: HALLMARK\_EPITHELIAL\_MESENCHYMAL\_TRANSITION      
 Profile of the Running ES Score & Positions of GeneSet Members on the Rank Ordered List

  

| PROBE | GENE SYMBOL | GENE\_TITLE | RANK IN GENE LIST | RANK METRIC SCORE | RUNNING ES | CORE ENRICHMENT || 1 | TAGLN |  |  | 43 | 1.279 | 0.0094 | Yes |
| 2 | MCM7 |  |  | 45 | 1.273 | 0.0292 | Yes |
| 3 | PTX3 |  |  | 139 | 0.981 | 0.0214 | Yes |
| 4 | MFAP5 |  |  | 140 | 0.978 | 0.0368 | Yes |
| 5 | FBN1 |  |  | 143 | 0.972 | 0.0516 | Yes |
| 6 | GADD45B |  |  | 146 | 0.965 | 0.0663 | Yes |
| 7 | TPM2 |  |  | 150 | 0.957 | 0.0806 | Yes |
| 8 | COL4A1 |  |  | 168 | 0.929 | 0.0910 | Yes |
| 9 | PLAUR |  |  | 185 | 0.893 | 0.1011 | Yes |
| 10 | INHBA |  |  | 196 | 0.876 | 0.1124 | Yes |
| 11 | QSOX1 |  |  | 204 | 0.870 | 0.1243 | Yes |
| 12 | MYL9 |  |  | 247 | 0.827 | 0.1269 | Yes |
| 13 | TPM1 |  |  | 259 | 0.805 | 0.1368 | Yes |
| 14 | COL4A2 |  |  | 264 | 0.795 | 0.1483 | Yes |
| 15 | COL7A1 |  |  | 285 | 0.775 | 0.1555 | Yes |
| 16 | COL6A3 |  |  | 291 | 0.768 | 0.1664 | Yes |
| 17 | COL1A1 |  |  | 294 | 0.761 | 0.1779 | Yes |
| 18 | GLIPR1 |  |  | 296 | 0.758 | 0.1895 | Yes |
| 19 | SERPINE1 |  |  | 299 | 0.756 | 0.2009 | Yes |
| 20 | VEGFC |  |  | 316 | 0.740 | 0.2086 | Yes |
| 21 | EDIL3 |  |  | 331 | 0.727 | 0.2165 | Yes |
| 22 | IGFBP4 |  |  | 401 | 0.678 | 0.2100 | Yes |
| 23 | CTHRC1 |  |  | 449 | 0.651 | 0.2085 | Yes |
| 24 | NT5E |  |  | 481 | 0.637 | 0.2108 | Yes |
| 25 | FLNA |  |  | 483 | 0.637 | 0.2206 | Yes |
| 26 | DAB2 |  |  | 491 | 0.631 | 0.2288 | Yes |
| 27 | FSTL1 |  |  | 495 | 0.630 | 0.2379 | Yes |
| 28 | FSTL3 |  |  | 545 | 0.605 | 0.2352 | Yes |
| 29 | COL12A1 |  |  | 554 | 0.601 | 0.2427 | Yes |
| 30 | COL5A1 |  |  | 556 | 0.599 | 0.2519 | Yes |
| 31 | MEST |  |  | 563 | 0.593 | 0.2597 | Yes |
| 32 | SDC4 |  |  | 583 | 0.585 | 0.2642 | Yes |
| 33 | NTM |  |  | 592 | 0.582 | 0.2713 | Yes |
| 34 | THBS1 |  |  | 624 | 0.568 | 0.2725 | Yes |
| 35 | TNFRSF12A |  |  | 629 | 0.566 | 0.2804 | Yes |
| 36 | IL32 |  |  | 635 | 0.563 | 0.2881 | Yes |
| 37 | DKK1 |  |  | 709 | 0.530 | 0.2782 | Yes |
| 38 | MATN3 |  |  | 749 | 0.517 | 0.2766 | Yes |
| 39 | BASP1 |  |  | 802 | 0.497 | 0.2714 | Yes |
| 40 | LOXL2 |  |  | 838 | 0.488 | 0.2704 | Yes |
| 41 | SNAI2 |  |  | 842 | 0.486 | 0.2773 | Yes |
| 42 | LOX |  |  | 843 | 0.486 | 0.2849 | Yes |
| 43 | LRP1 |  |  | 856 | 0.483 | 0.2895 | Yes |
| 44 | MATN2 |  |  | 861 | 0.482 | 0.2961 | Yes |
| 45 | GADD45A |  |  | 867 | 0.480 | 0.3024 | Yes |
| 46 | SDC1 |  |  | 872 | 0.479 | 0.3090 | Yes |
| 47 | RHOB |  |  | 881 | 0.476 | 0.3145 | Yes |
| 48 | COL6A2 |  |  | 1021 | 0.437 | 0.2866 | No |
| 49 | TNFAIP3 |  |  | 1070 | 0.424 | 0.2813 | No |
| 50 | BGN |  |  | 1105 | 0.416 | 0.2794 | No |
| 51 | ITGA2 |  |  | 1114 | 0.415 | 0.2839 | No |
| 52 | COL11A1 |  |  | 1163 | 0.404 | 0.2783 | No |
| 53 | PVR |  |  | 1291 | 0.380 | 0.2525 | No |
| 54 | PLOD1 |  |  | 1292 | 0.380 | 0.2585 | No |
| 55 | ITGB3 |  |  | 1325 | 0.374 | 0.2564 | No |
| 56 | COL5A2 |  |  | 1362 | 0.366 | 0.2532 | No |
| 57 | CDH11 |  |  | 1440 | 0.349 | 0.2395 | No |
| 58 | TPM4 |  |  | 1578 | 0.328 | 0.2104 | No |
| 59 | PCOLCE |  |  | 1677 | 0.313 | 0.1909 | No |
| 60 | FGF2 |  |  | 1679 | 0.313 | 0.1955 | No |
| 61 | JUN |  |  | 1725 | 0.307 | 0.1891 | No |
| 62 | VCAN |  |  | 1793 | 0.299 | 0.1771 | No |
| 63 | SLIT2 |  |  | 1873 | 0.289 | 0.1619 | No |
| 64 | FERMT2 |  |  | 2003 | 0.272 | 0.1340 | No |
| 65 | CAPG |  |  | 2029 | 0.269 | 0.1320 | No |
| 66 | P3H1 |  |  | 2031 | 0.269 | 0.1360 | No |
| 67 | SERPINH1 |  |  | 2146 | 0.258 | 0.1116 | No |
| 68 | COL5A3 |  |  | 2176 | 0.254 | 0.1083 | No |
| 69 | MMP14 |  |  | 2295 | -0.258 | 0.0829 | No |
| 70 | CXCL12 |  |  | 2350 | -0.264 | 0.0736 | No |
| 71 | GJA1 |  |  | 2366 | -0.266 | 0.0741 | No |
| 72 | CAP2 |  |  | 2406 | -0.272 | 0.0686 | No |
| 73 | EFEMP2 |  |  | 2654 | -0.309 | 0.0118 | No |
| 74 | MMP2 |  |  | 2762 | -0.328 | -0.0098 | No |
| 75 | FBN2 |  |  | 3186 | -0.425 | -0.1087 | No |
| 76 | PDLIM4 |  |  | 3220 | -0.433 | -0.1102 | No |
| 77 | PMEPA1 |  |  | 3390 | -0.494 | -0.1446 | No |
| 78 | FZD8 |  |  | 3399 | -0.498 | -0.1387 | No |
| 79 | GAS1 |  |  | 3415 | -0.506 | -0.1345 | No |
| 80 | VEGFA |  |  | 3532 | -0.560 | -0.1547 | No |
| 81 | SAT1 |  |  | 3659 | -0.653 | -0.1759 | No |
| 82 | ID2 |  |  | 3664 | -0.654 | -0.1666 | No |
| 83 | HTRA1 |  |  | 3741 | -0.719 | -0.1742 | No |
| 84 | PRRX1 |  |  | 3758 | -0.737 | -0.1666 | No |
| 85 | PFN2 |  |  | 3841 | -0.832 | -0.1740 | No |
| 86 | MGP |  |  | 3883 | -0.908 | -0.1699 | No |
| 87 | FBLN1 |  |  | 3909 | -0.965 | -0.1610 | No |
| 88 | SCG2 |  |  | 3975 | -1.137 | -0.1593 | No |
| 89 | COL1A2 |  |  | 3988 | -1.187 | -0.1436 | No |
| 90 | CDH2 |  |  | 3998 | -1.233 | -0.1265 | No |
| 91 | DPYSL3 |  |  | 4012 | -1.349 | -0.1085 | No |
| 92 | TNC |  |  | 4039 | -1.549 | -0.0906 | No |
| 93 | CADM1 |  |  | 4042 | -1.568 | -0.0664 | No |
| 94 | IGFBP3 |  |  | 4058 | -1.726 | -0.0429 | No |
| 95 | IGFBP2 |  |  | 4094 | -3.345 | 0.0010 | No |
Table: GSEA details [plain text format]

  

Fig 2: HALLMARK\_EPITHELIAL\_MESENCHYMAL\_TRANSITION: Random ES distribution      
 Gene set null distribution of ES for **HALLMARK\_EPITHELIAL\_MESENCHYMAL\_TRANSITION**
